# The association between dietary intake of macro- and micronutrients and multimorbidity: a cross-sectional study in Cyprus

**DOI:** 10.1017/jns.2023.102

**Published:** 2023-11-24

**Authors:** Maria Kyprianidou, Stavri Chrysostomou, Kosmia Andreou, Agni Alexandrou, Rafaella Panagiotou, Costas A. Christophi, Konstantinos Giannakou

**Affiliations:** 1 Cyprus International Institute for Environmental and Public Health, Cyprus University of Technology, Limassol, Cyprus; 2 Department of Health Sciences, School of Sciences, European University Cyprus, Nicosia, Cyprus; 3 Department of Life Sciences, School of Sciences, European University Cyprus, Nicosia, Cyprus

**Keywords:** Chronic diseases, Cyprus, Diet, Multimorbidity, Nutrition

## Abstract

Research on the link between diet and multimorbidity is scarce, despite significant studies investigating the relationship between diet and individual chronic conditions. This study examines the association of dietary intake of macro- and micronutrients with multimorbidity in Cyprus's adult population. It was conducted as a cross-sectional study, with data collected using a standardised questionnaire between May 2018 and June 2019. The questionnaire included sociodemographic information, anthropometrics, medical history, dietary habits, sleep quality, smoking habits, and physical activity. The participants were selected using a stratified sampling method from adults residing in the five government-controlled municipalities of the Republic of Cyprus. The study included 1137 adults with a mean age of 40⋅8 years, of whom 26 % had multimorbidity. Individuals with multimorbidity consumed higher levels of sodium (*P* = 0⋅009) and vitamin A (*P* = 0⋅010) compared to those without multimorbidity. Additionally, higher fibre and sodium intake were also observed in individuals with at least one chronic disease of the circulatory system or endocrine system, compared to those with no chronic diseases in these systems (*P* < 0⋅05). Logistic regression models revealed that individuals with ≥2 chronic diseases compared to 0 or 1 chronic disease had higher fat intake (OR = 1⋅06, 95 % CI: 1⋅02, 1⋅10), higher iron intake (OR = 1⋅05, 95 % CI: 1⋅01, 1⋅09), lower mono-unsaturated fat intake (OR = 0⋅91, 95 % CI: 0⋅86, 0⋅96), and lower zinc intake (OR = 0⋅98, 95 % CI: 0⋅96, 0⋅99). Future research should replicate these results to further explore the intricate relationships between nutrient intake and multimorbidity. Our study's findings suggest that specific dietary components may contribute to preventing and managing multimorbidity.

## Introduction

Multimorbidity is defined as the co-occurrence of two or more chronic diseases.^(1,2)^ It is a common condition affecting a significant proportion of the adult population of all ages.^(3,4)^ Multimorbidity prevalence varies from 10 % to over 90 % among studies, mainly due to the use of different definitions, data collection methods, sociodemographic characteristics, and other factors.^(5)^ However, the overall prevalence of multimorbidity in adults has been estimated to be 18–30 %.^(3,4)^ According to recent data, the prevalence of multimorbidity is higher in older people, varying between 55 and 98 %.^(6)^

Multimorbidity is linked to the rise in life expectancy since a longer lifespan increases the chances of developing multiple chronic diseases. According to the World Health Organization (WHO), life expectancy is projected to increase to 78 years for males and 85 years for females by 2030, up from 70⋅8 and 76, respectively.^(7)^ Consequently, the prevalence of multimorbidity will increase even more,^(8,9)^ making it a global health priority.^(5)^ The 2013–2020 WHO Global Action Plan for the Prevention and Control of Non-Communicable Diseases (NCDs) aims to prevent and manage NCDs and the related risk factors, including smoking, low levels of physical activity, obesity, unhealthy diet, and increased alcohol consumption.^(10,11)^ Therefore, preventing and managing NCDs would help curb the burden of multimorbidity worldwide.

Many factors have been shown to be associated with multimorbidity, such as sociodemographic factors (e.g. sex, age, and educational level), socio-economic status, weight, physical activity, sleep, and diet.^(6,12–19)^ Several epidemiological studies have focused on the effect of diet on multimorbidity and related chronic diseases. According to the 2020 WHO fact sheet, maintaining a healthy diet is a crucial factor in protecting against a wide range of NCDs. Key recommendations for a healthy diet include balancing energy intake with energy expenditure to maintain a healthy body weight, ensuring that total fat intake does not exceed 30 % of total energy intake, keeping saturated fat intake below 10 % of total energy intake, limiting the intake of trans-fats to less than 1 % of total energy intake, restricting free sugars intake to less than 5 % of total energy intake, and limiting salt intake to no more than 5 g per day to prevent hypertension and other cardiovascular diseases.^(20)^ Indeed, a healthy diet with a higher consumption of fruits and vegetables, whole grain products, fish, and nuts, and a lower consumption of red meat and sweets decreases the risk of developing several chronic diseases, including obesity, stroke, heart disease, hypertension, hyperlipidaemia, and cancer.^(11,21,22)^ However, research related to the effect of specific dietary components on multimorbidity is scarce.

A recent epidemiological study in Cyprus that investigated multimorbidity in a representative sample of the adult population indicated that the age and sex standardised prevalence of multimorbidity was 28⋅6 %,^(23)^ which is close to that of other countries, such as Scotland (23⋅2 %),^(24)^ Brazil (23⋅6 %),^(12)^ Serbia (26⋅9 %),^(25)^ and Australia (25⋅5 %).^(26)^ Of all countries in the European Union, Cyprus ranks as one of those with the highest life expectancy (82⋅2 years)^(27)^. Given the aging population of Cyprus, and the expected increase in life expectancy, the high prevalence of overweight and obese people,^(28)^ the high prevalence of smoking,^(29)^ and the association of multimorbidity increases with these conditions, it is expected that the prevalence of multimorbidity will increase further in the future.^(9)^

Furthermore, it has been reported that higher adherence to the Mediterranean diet is associated with lower odds of multimorbidity.^(18)^ Further examinations within the same study showed that good quality of sleep also reduces the risk of multimorbidity.^(14)^ Thus, it is important to examine closely modifiable factors associated with multimorbidity. At the same time, it would be important to investigate the potential association of the consumption of specific nutrients and multimorbidity. To the best of our knowledge, there is limited data on the association of dietary intake of macro- and micronutrients with multimorbidity. Thus, the objective of this study was to examine the relationship between dietary intake (macro- and micronutrients) and multimorbidity in Cyprus's adult population. The effect of specific nutrients in multimorbidity is important to inform targeted dietary interventions for the prevention of multimorbidity.

## Materials and methods

### Setting and sampling

The study was cross-sectional. Assuming a 95 % confidence level and a 5 % margin of error, we calculated that a sample size of 1145 participants was required for the study, based on an estimated true prevalence of multimorbidity in the population of 30 %. The population of the study comprised of adult males and females (≥18 years old) who were residents of the five government-controlled municipalities of the Republic of Cyprus (Nicosia, Limassol, Larnaca, Paphos, and Ammochostos). The data collection took place in various public locations, including restaurants, malls, cafes, and village squares, as well as in private residences throughout Cyprus. This process took place over the course of 1 year, from May 2018 to June 2019, with face-to-face interviews conducted by trained investigators. To mitigate potential biases during participant recruitment, we implemented an unrestricted and as-random-as-possible sampling approach. We selected public places across all geographical areas of Cyprus, ensuring representation of individuals of all ages (over 18 years) and collecting data at various times of the day and all days of the week, including weekends. We aimed to gather data from both urban and rural regions within the five geographical areas of Cyprus in a representative manner. Additionally, we avoided locations with potentially high number of people with chronic diseases, such as establishments near hospitals and nursing homes. Our stratified sampling was used to ensure that the study sample matched the population of Cyprus in three main demographic characteristics, namely age, sex, and residency. To validate the sample's similarity to the Cypriot population, we performed chi-square goodness-of-fit tests, comparing the distribution of the sample size for each key characteristic with the corresponding distribution in the overall Cypriot population. The results indicated that our selected study sample closely mirrored the general population in terms of region, age, and sex distributions, with all *P*-values exceeding 0⋅05. The response rate for our study was 90 %. More information regarding sampling and other aspects of the study can be found elsewhere.^(23)^

### Participants’ characteristics

The data collection was performed using a standardised questionnaire that included questions on sociodemographic characteristics, anthropometric characteristics, medical history, dietary habits, and lifestyle characteristics (i.e. quality of sleep, smoking, and physical activity).

### Sociodemographic characteristics

Sex was recorded as males or females and age was reported in years and categorised in four groups based on the age categorisation of the Statistical Service of Cyprus:^(30)^ 18–24 years old, 25–44 years old, 45–64 years old, and more than 65 years old. Educational level was categorised in three categories as commonly used in Cyprus, namely, primary education (participants who completed only primary school — <7 years of schooling); (ii) secondary education (participants who completed middle or high school — 7–12 years of schooling); (iii) higher education (participants who have a university degree — >12 years of schooling). Marital status was classified as married, unmarried, and divorced/widowed. Job status was recorded as a private or state employee, unemployed, freelance, or retired.

### Anthropometric characteristics

Self-reported weight and height were provided in kilograms and metres, respectively. The Body Mass Index (BMI) was calculated as weight divided by height squared and categorised as obese with BMI >29⋅9 kg/m^2^, overweight with BMI 25–29⋅9 kg/m^2^, normal with BMI 18⋅5–24⋅9 kg/m^2^, and underweight with BMI <18⋅5 kg/m^2^.^(31)^

### Medical history

The question ‘Have you ever been diagnosed by a physician with any of the following chronic diseases? Choose all that apply’ was used to collect the medical history of the participants. It considered 47 chronic diseases according to the International Classification of Diseases, 10^th^ Revision (ICD-10) of all circulatory, endocrine, digestive/excretory, nervous, respiratory, immune, skeletal/muscular, renal/urinary, reproductive systems, as well as neoplasms (Supplementary File 1). The list of 47 diseases or conditions was compiled from the existing literature and epidemiological studies that have previously investigated the prevalence of multimorbidity in various populations. To ensure data accuracy, we collected medical history through face-to-face interviews. This approach clarified that only chronic diseases diagnosed by a doctor should be selected. Additionally, it allowed us to provide any necessary clarifications to participants, ensuring the reliability of their responses. Multimorbidity was defined as the co-occurrence of two or more chronic diseases.^(32)^

### Dietary habits

A detailed validated semi-quantitative food frequency questionnaire (FFQ) was used to obtain the dietary habits of the participants.^(33)^ The questionnaire collected information on the consumption of 11 food groups, namely non-refined cereals, fruits, vegetables, legumes/pulses, potatoes, fish, meat and meat products, poultry, full-fat dairy products, olive oil, and alcohol intake (measured in wine glasses, equivalent to 100 mL, and quantified by ethanol intake in grams per day). To assess daily energy and nutrient intake, after completing all FFQs, we converted the quantities of food items into daily intake measurements. Thereafter, the daily intake of each food item was entered into a nutrition software for the assessment of energy (kcal), carbohydrates (CHO) (g), sugar (g), fibre (g), protein (g), fat (g), saturated fat (SF) (g), poly-unsaturated fat (PUFA) (g), mono-unsaturated fat (MUFA) (g), iron (mg), calcium (Ca) (mg), sodium (Na) (mg), potassium (K) (mg), vitamin A (mg), vitamin E (mg), and zinc (Zn) (mg) daily intake. The analysis of all data pertaining to the mentioned nutrients was conducted using the dietary analysis software, SNPRO Nutrition Software, developed by Cheapsoft Softwares in 2017. The SNPRO Nutrition Software is using the USDA Food Database which includes more than 8600 different food items for the analysis of more than 100 nutrients. Moreover, adherence to the Mediterranean diet was evaluated using the MedDietScore,^(34)^ which ranges from 0 to 55, with higher values indicating greater adherence to the Mediterranean diet. The tertiles of adherence to a Mediterranean diet were defined as follows: low: MedDietScore ≤13; moderate: MedDietScore 14–17; and high: MedDietScore ≥18. In addition, dietary supplements were assessed in three categories: vitamins, proteins, and creatine. Participants also had the option to specify ‘Other’ supplements, if needed. To ensure an accurate dietary data collection over the past 30 days, the data collection process spanned over 1 year, with the same number of questionnaires collected each month. This approach allowed us to maintain a uniform distribution across all seasons, including holiday periods such as summer, Christmas, Easter, and religious fasting periods.

### Lifestyle characteristics

Quality of sleep was evaluated using the Pittsburgh Sleep Quality Index (PSQI) questionnaire, in Greek, as kindly provided by the University of Pittsburgh. The PSQI consists of 19 self-rated questions each scored on a 0–3 scale. The PSQI score has a maximum score of 21, with higher scores indicating worse sleep quality. Smoking was evaluated using a question about current smoking status (i.e. current smoker and non-current smoker). Physical activity status was categorised as physically inactive and physically active.

### Ethics approval

This study was conducted according to the guidelines laid down in the Declaration of Helsinki and all procedures involving human subjects were approved by the Cyprus National Bioethics Committee (CNBC) (EEΒK EΠ 2018⋅01⋅123). Verbal informed consent was obtained from all subjects. Verbal consent was witnessed and formally recorded.

### Statistical analysis

The Shapiro-Wilk normality test was used to examine the distribution of the continuous variables. Continuous variables with normal distribution were presented using mean ± standard deviation (sd) while categorical variables were presented as absolute (*n*) and relative (%) frequency. Student's *t*-test and ANOVA were used for the comparison of the continuous variables among the groups of the categorical variables. The χ^2^ test of independence was used to evaluate the association between categorical variables. Hierarchical logistic regression modelling was performed to evaluate the significance of nutritional intake on the number of chronic diseases (≥2 chronic diseases *v.* 0 or 1 chronic disease). We first applied the crude model (*Model 1*), then we adjusted for sociodemographic characteristics (age, sex, geographical area, residency, marital status, educational level, job, and salary status) (*Model 2*), and finally we adjusted for lifestyle characteristics (exercise, smoking, Mediterranean Diet adherence, quality of sleep, and BMI) (*Model 3*). All statistical tests performed were two-sided, with the statistical significance level set at α = 0⋅05. Statistical analysis was conducted using STATA 14⋅0 (Stata Corp, College Station, TX, USA).

## Results

### Participants’ characteristics overall and by multimorbidity groups

A total of 1137 adults participated in the study after excluding three participants who had more than 20 % of missing data in the FFQ section of the questionnaire. The mean age of the participants was 40⋅8 ± 16⋅9 years old, and 14⋅7 % of the participants were 18–24 years old, 46⋅0 % were 25–44 years old, 27⋅5 % were 45–64 years old, and 11⋅8 % were 65 years old or older. More than half (56⋅4 %) were females and 43⋅6 % were males. Most of the participants were residents of Nicosia (43⋅3 %) and urban areas (76⋅2 %), were married (54⋅3 %), had completed higher education (64⋅4 %), had an average income (46⋅7 %), and were private employees (39⋅7 %). Individuals in the multimorbidity group were significantly older than the people in the non-multimorbidity group (*P* < 0⋅001). The age differences were more pronounced among males, unmarried individuals, those who completed higher education, and private employees (all *P*-values < 0⋅001). We also found that a larger percentage of individuals with multimorbidity were current smokers (*P* = 0⋅017), physically inactive (*P* < 0⋅001), had a poorer quality of sleep (*P* < 0⋅001), and were obese (*P* < 0⋅001) (Table 1).

**Table 1.** Sociodemographic and lifestyle characteristics overall, and by multimorbidity

	Overall (*N* 1137)	Multimorbidity		*P*-value
		No (*N* 844)	Yes (*N* 293)	
Mean age (sd)	40⋅8 ± 16⋅9	36⋅6 ± 14⋅2	52⋅9 ± 18⋅0	**<0⋅001**^a^
Age group, *N* (%)				
18–24	167 (14⋅7)	154 (92⋅2)	13 (7⋅8)	**<0⋅001**^b^
25–44	523 (46⋅0)	439 (83⋅9)	84 (16⋅1)	
45–64	313 (27⋅5)	210 (67⋅1)	103 (32⋅9)	
65+	134 (11⋅8)	41 (30⋅6)	93 (69⋅4)	
Sex, *N* (%)				
Males	496 (43⋅6)	384 (77⋅4)	112 (22⋅6)	**0⋅031**^b^
Females	641 (56⋅4)	460 (71⋅8)	181 (28⋅2)	
Geographical area, *N* (%)				
Nicosia	492 (43⋅3)	361 (73⋅4)	131 (23⋅6)	0⋅172^b^
Limassol	310 (27⋅3)	221 (71⋅3)	89 (28⋅7)	
Larnaca	171 (15⋅0)	135 (79⋅0)	36 (21⋅0)	
Paphos	113 (10⋅0)	91 (80⋅5)	22 (19⋅5)	
Ammochostos	50 (4⋅4)	35 (70⋅0)	15 (30⋅0)	
Residency, *N* (%)				
Urban	862 (76⋅2)	634 (73⋅5)	228 (26⋅5)	0⋅384^b^
Rural	269 (23⋅8)	205 (76⋅2)	64 (23⋅8)	
Marital status, *N* (%)				
Married	614 (54⋅3)	420 (68⋅4)	194 (31⋅6)	**<0⋅001**^b^
Unmarried	421 (37⋅2)	371 (88⋅1)	50 (11⋅9)	
Divorced/Widowed	96 (8⋅5)	50 (52⋅1)	46 (47⋅9)	
Educational level, *N* (%)				
Primary education	66 (5⋅8)	18 (27⋅3)	48 (72⋅7)	**<0⋅001**^b^
Secondary education	337 (29⋅8)	250 (74⋅2)	87 (25⋅8)	
Higher education	728 (64⋅4)	571 (78⋅4)	157 (21⋅6)	
Annual income, *N* (%)				
Low (≤€6500)	241 (21⋅3)	188 (78⋅0)	53 (22⋅0)	0⋅163^b^
Middle (€6500–19 500)	561 (46⋅7)	417 (74⋅3)	144 (25⋅7)	
High (≥€19 501)	327 (29⋅0)	232 (71⋅0)	95 (29⋅0)	
Job status, *N* (%)				
Private employee	430 (39⋅7)	357 (83⋅0)	73 (17⋅0)	**<0⋅001**^b^
State employee	218 (20⋅2)	159 (72⋅9)	59 (27⋅1)	
Freelance	100 (9⋅2)	81 (81⋅0)	19 (19⋅0)	
Unemployed	205 (19⋅0)	169 (82⋅4)	36 (17⋅6)	
Retired	129 (11⋅9)	38 (29⋅5)	91 (70⋅5)	
Smoking status, *N* (%)				
Non-smoker	729 (64⋅5)	556 (76⋅4)	172 (23⋅6)	**0⋅017**^b^
Current smoker	402 (35⋅5)	281 (69⋅9)	121 (30⋅1)	
Physical activity level, *N* (%)				
Physical inactive	591 (52⋅3)	406 (68⋅8)	184 (31⋅2)	**<0⋅001**^b^
Physical active	539 (47⋅7)	431 (80⋅0)	108 (20⋅0)	
Mediterranean Diet adherence, *N* (%)				
Low (≤13)	379 (33⋅3)	271 (71⋅5)	108 (28⋅5)	0⋅153^b^
Moderate (14–17)	412 (36⋅3)	304 (73⋅8)	108 (26⋅2)	
High (≥18)	346 (30⋅4)	269 (77⋅7)	77 (22⋅3)	
Quality of sleep, *N* (%)				
Good (≤ 5)	692 (60⋅9)	549 (79⋅3)	143 (20⋅7)	**<0⋅001**^b^
Poor (>5)	445 (39⋅1)	295 (66⋅3)	150 (33⋅7)	
BMI, *N* (%)				
Normal	565 (50⋅5)	453 (80⋅2)	112 (19⋅8)	**<0⋅001**^b^
Underweight	42 (3⋅7)	37 (87⋅1)	5 (11⋅9)	
Overweight	361 (32⋅3)	256 (70⋅9)	105 (29⋅1)	
Obese	151 (13⋅5)	84 (56⋅0)	66 (44⋅0)	

CHO, carbohydrates; Sat, saturated; PUFA, poly-unsaturated fat; MUFA, mono-unsaturated fat; Fe, iron; Ca, calcium; Na, sodium; K, potassium; Vit. A, vitamin A; Vit E., vitamin E; sd, standard deviation; *N* (%), frequency (percentage).

a Differences between groups tested using t-test.

b Differences between groups were tested using χ^2^-test.

Bold values indicate statistically significant associations (*P* < 0⋅05).

### Nutritional intake and multimorbidity

The mean (±sd) values for various nutrients were as follows: energy intake, 2526⋅8 (±7717⋅7); carbohydrates (CHO), 252⋅5 (±153⋅6); sugars, 4⋅4 (±8⋅4); fibre, 24⋅2 (±18⋅4); protein, 198⋅9 (±123⋅1); fat, 74⋅0 (±53⋅2); saturated fat (sat), 29⋅1 (±24⋅4); PUFA, 7⋅2 (±5⋅4); MUFA, 30⋅1 (±22⋅2); iron, 25⋅2 (±24⋅7); calcium (Ca), 1646⋅9 (±1383⋅5); sodium (Na), 2278⋅0 (±1811⋅9); potassium (K), 10574⋅83 (±22⋅2); vitamin A, 1460⋅1 (±2687⋅5); vitamin E, 0⋅5 (±1⋅4); and zinc, 22⋅2 (±63⋅4) (Table 2). Out of the total 1137 participants, 844 (74⋅2 %) did not have multimorbidity, while 293 (25⋅8 %) had multimorbidity.

**Table 2.** Dietary intake of macro- and micronutrients overall, and by multimorbidity

	Overall (*N* 1137)	Multimorbidity		*P*-value^a^
		No (*N* 844)	Yes (*N* 293)	
Energy	2526⋅8 ± 7717⋅7	2622⋅9 ± 8907⋅9	2250⋅5 ± 1614⋅2	0⋅4770
CHO	252⋅5 ± 153⋅6	249⋅8 ± 137⋅4	260⋅2 ± 192⋅8	0⋅3192
Sugars	4⋅4 ± 8⋅4	4⋅2 ± 6⋅4	4⋅9 ± 12⋅4	0⋅2539
Fibres	24⋅2 ± 18⋅4	23⋅9 ± 17⋅7	25⋅1 ± 20⋅2	0⋅3464
Protein	198⋅9 ± 123⋅1	202⋅6 ± 122⋅2	188⋅5 ± 125⋅4	0⋅1085
Fat	74⋅0 ± 53⋅2	73⋅2 ± 47⋅1	76⋅4 ± 67⋅7	0⋅3644
Sat	29⋅1 ± 24⋅4	29⋅5 ± 24⋅1	27⋅9 ± 25⋅3	0⋅3405
PUFA	7⋅2 ± 5⋅4	7⋅2 ± 4⋅8	7⋅4 ± 6⋅9	0⋅4703
MUFA	30⋅1 ± 22⋅2	29⋅8 ± 19⋅6	30⋅9 ± 28⋅5	0⋅4851
Iron	25⋅2 ± 24⋅7	25⋅5 ± 25⋅2	24⋅5 ± 23⋅3	0⋅5301
Ca	1646⋅9 ± 1383⋅5	1652⋅0 ± 1364⋅0	1632⋅2 ± 1440⋅4	0⋅8331
Na	2278⋅0 ± 1811⋅9	2195⋅5 ± 1408⋅9	2515⋅4 ± 2638⋅2	**0⋅0092**
K	10574⋅83 ± 22⋅2	9999⋅7 ± 10381⋅9	12229⋅5 ± 28488⋅7	0⋅0534
Vit. A	1460⋅1 ± 2687⋅5	1336⋅0 ± 2281⋅4	1820⋅2 ± 3597⋅6	**0⋅0100**
Vit. E	0⋅5 ± 1⋅4	0⋅4 ± 1⋅4	0⋅5 ± 1⋅2	0⋅5235
Zinc	22⋅2 ± 63⋅4	23⋅7 ± 71⋅8	17⋅9 ± 27⋅1	0⋅1783

CHO, carbohydrates; Sat, saturated; PUFA, poly-unsaturated fat; MUFA, mono-unsaturated fat; Ca, calcium; Na, sodium; K, potassium; Vit. A, vitamin A; Vit E., vitamin E.

a Differences between groups were tested using t-test; Data are presented as mean ± standard deviation.

Bold values indicate statistically significant associations (*P* < 0⋅05).

### Nutritional intake by multimorbidity groups and human systems

We only found statistically significant differences between multimorbidity groups in relation to Na (*P* = 0⋅009) and vitamin A (*P* = 0⋅010) intake. Specifically, individuals with multimorbidity consumed more Na and Vitamin A compared to the participants without multimorbidity (Table 2).

In addition, individuals who had at least one chronic disease of the circulatory system had a higher intake of fibre (*P* = 0⋅018), Na (*P* = 0⋅012), and vitamin A (*P* = 0⋅001) compared to those who did not have any chronic diseases of the circulatory system. In addition, we found a higher intake of fiber (*P* = 0⋅045), MUFA (*P* = 0⋅035), and Na (*P* = 0⋅014) in individuals who had at least one chronic disease of the endocrine system compared to those who had not chronic diseases of the endocrine system. Similarly, a higher mean intake of sugars was identified in participants with at least one chronic disease of the skeletal/muscular system (mean intake = 7⋅6 g) compared to the participants without a chronic disease of the skeletal/muscular system (mean intake = 4⋅3 g) (*P* = 0⋅027). On the other hand, a higher intake of protein was identified in participants without a chronic disease of the respiratory system compared to those with at least one chronic disease of the respiratory system (*P* = 0⋅038). Larger intakes of fat (*P* = 0⋅033), SF (*P* = 0⋅018), Na (*P* = 0⋅001), and Zn (*P* = 0⋅029) were reported in participants without a chronic disease of the renal/urinary system compared to those with at least one chronic disease of the renal/urinary system (Supplementary Table 1).

### Nutritional intake by sociodemographic

Supplementary Table 2 presents the participants’ nutritional intake based on their sociodemographic characteristics. We found that individuals aged 18–24 years old had a higher intake of sugar (*P* < 0⋅001), protein (*P* < 0⋅001), SF (*P* < 0⋅001), and Zn (*P* = 0⋅025) compared to other age groups. A statistically significant difference was only observed in sugar intake between males and females, with the largest intake observed in females (*P* = 0⋅040). Residents of rural areas had a lower intake of CHO (*P* < 0⋅001), protein (*P* < 0⋅001), Ca (*P* < 0⋅001), Na (*P* < 0⋅001), and K (*P* = 0⋅025) compared to residents of urban areas. Divorced or widowed individuals had a higher intake of daily energy and vitamins, while unmarried participants had a higher intake of protein and SF (*P*-values < 0⋅05). Retired individuals had a lower intake of protein (*P* < 0⋅001) and SF (*P* < 0⋅001).

### Nutritional intake by lifestyle characteristics

We found a lower intake of sugar (*P* = 0⋅031), fat (*P* = 0⋅005), SF (*P* = 0⋅004), and PUFA (*P* = 0⋅045) in current smokers compared to non-smokers (Supplementary Table 3). In terms of Mediterranean diet adherence, we observed higher intakes of CHO, fibres, protein, fat, SF, PUFA, MUFA, iron, Ca, and Na among participants who were classified as having high adherence to the Mediterranean diet, compared to those with low or moderate adherence (all *P*-values < 0⋅001). In terms of sleep quality groups, we found a statistically significant difference only in K intake (*P* = 0⋅029). Additionally, higher intake of mono-unsaturated fat was reported in underweight individuals compared to other BMI categories (*P* < 0⋅001) (Supplementary Table 3).

### Associations of nutritional intake and multimorbidity

Based on the hierarchical logistic regression model, the presence of ≥2 chronic diseases in an individual compared to 0 or 1 chronic disease was associated with an increased fat intake (*P* = 0⋅016) and a lower MUFA intake (*P* = 0⋅006) (Model 1). After adjusting for sociodemographic characteristics (Model 2), the results showed that an increased fat intake (*P* = 0⋅001), iron intake (*P* = 0⋅019), and vitamin E intake (*P* = 0⋅048), as well as a lower MUFA intake (*P* = 0⋅001) and Ca intake (*P* = 0⋅038), were all associated with an increased probability of having two or more chronic diseases compared to 0 or 1 chronic disease. In the final model (Model 3), which further adjusted for lifestyle characteristics, it was found that the presence of ≥2 chronic diseases in an individual compared to 0 or 1 chronic disease was associated with an increased fat intake (OR = 1⋅06, 95 % CI: 1⋅02, 1⋅10), iron intake (OR = 1⋅05, 95 % CI: 1⋅01, 1⋅09), and lower MUFA intake (OR = 0⋅91, 95 % CI: 0⋅86, 0⋅96), and Zn intake (OR = 0⋅98, 95 % CI: 0⋅96, 0⋅99) (Table 3).

**Table 3.** Hierarchical logistic regression modelling for dietary intake on number of chronic diseases (≥2 chronic diseases *v.* 0 or 1 chronic disease)

	Model 1		Model 2		Model 3	
	OR (95 % CI)	*P*-value	OR (95 % CI)	*P*-value	OR (95 % CI)	*P*-value
Energy	1⋅00 (1⋅00, 1⋅00)	0⋅155	1⋅00 (1⋅00, 1⋅00)	0⋅068	1⋅00 (1⋅00, 1⋅00)	0⋅049
CHO	1⋅01 (1⋅00, 1⋅01)	0⋅102	1⋅01 (1⋅00, 1⋅02)	0⋅063	1⋅01 (1⋅00, 1⋅02)	0⋅052
Sugars	1⋅02 (0⋅98, 1⋅06)	0⋅282	1⋅04 (0⋅99, 1⋅09)	0⋅108	1⋅04 (0⋅99, 1⋅10)	0⋅084
Fibres	0⋅99 (0⋅97, 1⋅01)	0⋅387	0⋅98 (0⋅96, 1⋅01)	0⋅198	0⋅99 (0⋅96, 1⋅01)	0⋅288
Protein	1⋅00 (1⋅00, 1⋅00)	0⋅609	1⋅00 (1⋅00, 1⋅00)	0⋅455	1⋅00 (1⋅00, 1⋅00)	0⋅448
Fat	**1⋅04 (1⋅01, 1⋅07)**	**0⋅016**	**1⋅06 (1⋅02, 1⋅10)**	**0⋅001**	**1⋅06 (1⋅02, 1⋅10)**	**0⋅002**
Sat	0⋅98 (0⋅96, 1⋅01)	0⋅214	0⋅98 (0⋅96, 1⋅01)	0⋅297	0⋅99 (0⋅96, 1⋅02)	0⋅434
PUFA	1⋅00 (0⋅92, 1⋅10)	0⋅958	0⋅98 (0⋅89, 1⋅09)	0⋅740	0⋅99 (0⋅89, 1⋅10)	0⋅874
MUFA	**0⋅94 (0⋅90, 0⋅98)**	**0⋅006**	**0⋅91 (0⋅86, 0⋅96)**	**0⋅001**	**0⋅91 (0⋅86, 0⋅96)**	**0⋅001**
Iron	1⋅02 (0⋅99, 1⋅06)	0⋅147	**1⋅04 (1⋅01, 1⋅08)**	**0⋅019**	**1⋅05 (1⋅01, 1⋅09)**	**0⋅008**
Ca	1⋅00 (1⋅00, 1⋅00)	0⋅268	**0⋅99 (0⋅99, 1⋅00)**	**0⋅038**	**1⋅00 (1⋅00, 1⋅00)**	**0⋅040**
Na	1⋅00 (1⋅00, 1⋅00)	0⋅070	**1⋅00 (1⋅00, 1⋅00)**	**0⋅042**	**1⋅00 (1⋅00, 1⋅00)**	**0⋅032**
K	1⋅00 (1⋅00, 1⋅00)	0⋅397	1⋅00 (1⋅00, 1⋅00)	0⋅501	1⋅00 (1⋅00, 1⋅00)	0⋅478
Vitamin A	1⋅00 (1⋅00, 1⋅00)	0⋅517	1⋅00 (1⋅00, 1⋅00)	0⋅360	1⋅00 (1⋅00, 1⋅00)	0⋅253
Vitamin E	1⋅34 (0⋅95, 1⋅88)	0⋅098	**1⋅53 (1⋅00, 2⋅32)**	**0⋅048**	1⋅48 (0⋅96, 2⋅30)	0⋅079
Zinc	0⋅98 (0⋅97, 1⋅00)	0⋅078	0⋅98 (0⋅96, 1⋅00)	0⋅065	**0⋅98 (0⋅96, 0⋅99)**	**0⋅021**

CHO, carbohydrates; Sat, saturated; PUFA, poly-unsaturated fat; MUFA, mono-unsaturated fat; Ca, calcium; Na, sodium; K, potassium; Vit. A, vitamin A; Vit E., vitamin E; CI; Confidence Interval; Model 1, Crude Model; Model 2, Crude model adjusted for sociodemographic (age, sex, geographical area, residency, marital, educational, job and salary status); Model 3, Model 2 adjusted for lifestyle characteristics (exercise, smoking, Mediterranean Diet adherence, quality of sleep, BMI).

Bold values indicate statistically significant associations (*P* < 0⋅05).

### Food consumption

Supplementary Table 4 displays the food consumption patterns of the participants. The data indicates that most participants reported consuming 13–18 portions of non-refined cereals per week, 9–15 portions of fruits per week, 13–20 portions of vegetables per week, 3–4 portions of legumes per week, 9–12 portions of potatoes per week, 1–2 portions of fish per week, 4–5 portions of meat and meat products per week, 7–8 portions of poultry per week, and 21–28 portions of full-fat dairy products per week. Additionally, the majority of the respondents mentioned a daily consumption of olive oil and an average alcohol intake of 400 mL per day.

## Discussion

To the best of our knowledge, this is the first study to explore the association between dietary intake (macro- and micronutrients) and multimorbidity. The present study found that the presence of ≥2 chronic diseases *v.* 1 chronic disease is associated with a statistically significant increase in fat and iron intake and a significantly lower intake of MUFA and Zn. These intriguing findings indicate that dietary interventions focusing on reducing fat consumption while promoting the intake of MUFA and Zn may have promising implications for preventing or managing multimorbidity in individuals with multiple chronic diseases. Implementing targeted dietary strategies based on these associations has the potential to positively impact the overall health and well-being of individuals facing a higher burden of chronic conditions, contributing to improved disease management and enhanced quality of life.

Our findings show that having ≥2 chronic diseases *v.* 0 or 1 chronic condition is associated with a statistically significant increase in iron intake. Iron is an essential nutrient necessary for the production of haemoglobin, the protein in erythrocytes that carries oxygen to the body's tissues.^(35)^ However, when iron levels become too high, it can lead to oxidative stress and inflammation, damaging cells and contributing to the development of chronic diseases, such as neurodegenerative diseases, cancer, heart disease, and diabetes.^(36–38)^ The association between increased fat intake and multimorbidity, also observed in our study, is complex and involves several physiological mechanisms. High-fat diets can lead to increased levels of circulating lipids and insulin resistance, contributing to the development of metabolic disorders such as cardiovascular disease, type 2 diabetes, and certain cancers.^(39,40)^ Furthermore, high-fat diets have been linked to low-grade inflammation, oxidative stress, and changes in gut microbiota, all of which can contribute to the development of multiple chronic conditions.^(41,42)^ There is currently a lack of research studies that specifically examine the association between increased fat and iron intake and multimorbidity. This is an area that requires further investigation, as understanding the potential link between these two factors and the development of multiple chronic health conditions could have important implications for public health. Despite this gap, some studies have examined the association of red meat consumption and multimorbidity, as red meat contains high levels of fat and iron, and a previous overview of systematic reviews and meta-analyses suggested that overall, a high intake of processed meat is related to an increased risk of various cancers, type 2 diabetes, and cardiovascular disease incidence and mortality.^(43)^ However, it is important to note that these studies are observational in nature and cannot establish cause and effect, while it is essential to consider the quality of the study and the potential biases that may have influenced the results. More research is needed to fully understand the relationship between red meat consumption and multimorbidity, and to identify any potential links between the two.

Our findings show that having ≥2 chronic diseases *v.* 0 or 1 chronic condition is associated with a statistically significant lower intake of MUFA and Zn. The relationship between Zn and MUFA and multimorbidity is complex and not fully understood. Zn is an essential mineral that plays a critical role in many physiological processes, including immune function and DNA synthesis.^(44,45)^ On the other hand, MUFAs are considered to be a healthy type of fat that can help lower cholesterol levels, improve insulin sensitivity, impact positively on fat distribution, and reduce oxidative stress and inflammation.^(46–48)^ However, the specific relationship between Zn and MUFA intake and the development of multiple chronic conditions, particularly in the context of multimorbidity, is not well established and requires further research. Understanding the intricate interplay between Zn and MUFA intake and their impact on underlying biological processes, such as inflammation, oxidative stress, and immune function, could provide crucial insights for developing targeted dietary interventions aimed at mitigating the burden of multimorbidity and/or specific chronic diseases.

The findings of this study highlight the importance of considering specific nutrient intake in the development of multimorbidity. It is recommended that public health efforts focus on promoting healthy dietary patterns and nutrient intake to reduce the risk of multimorbidity. This could include education and awareness campaigns about the importance of a balanced diet, providing access to healthy food options, and encouraging physical activity. Our findings highlight the need for continued research in this area to better understand the relationship between diet, nutrient intake, and multimorbidity to develop evidence-based interventions to improve health outcomes for individuals with multiple chronic conditions. Further research could explore the optimal nutrient levels for individuals with multimorbidity, considering their individual needs and health status. The results of such research could inform the development of personalised nutrition recommendations for individuals with multiple chronic conditions. Lastly, interventional studies examining the impact of specific dietary interventions on multimorbidity risk and outcomes are needed to support the implementation of effective public health and clinical interventions.

This study investigates the relationship between dietary intake of macro- and micronutrients with multimorbidity in a nationally representative sample of both men and women in Cyprus. The dietary intake data was collected using a validated questionnaire and the presence of specific chronic diseases was recorded using standardised definitions. Despite the strengths of the study, such as the collection of detailed dietary data and information on many chronic diseases, there are also limitations to consider. It is important to acknowledge that self-report data collected from participants can be prone to bias due to social desirability. Participants may report their dietary intake in a more positive light, leading to underestimates of actual consumption. In addition, there is a possibility of misclassification bias due to the self-reported dietary intake and presence of chronic diseases, which could impact the accuracy and validity of the findings. Moreover, the analysis of nutritive values of each food item based on history can be misleading. Another limitation is that the study did not consider the severity of the chronic diseases included, as all were given equal weight in the calculation of multimorbidity. Also, although the study adjusted for potential confounders, the effect of any unmeasured factors, such as taking specific supplements or taking certain medications, on the observed association cannot be excluded. Furthermore, as the study is cross-sectional, it does not consider the time effect on multimorbidity and can only investigate associations between the groups of interest, not causal relationships. Finally, our study was limited by the potential influence of certain characteristics of the chronic diseases studied on participants’ food intake and nutritional status, highlighting them as potential confounding factors. However, the logistical challenge of obtaining comprehensive data on the dietary behaviours and nutritional status of individuals with diverse chronic conditions, given the multifaceted nature of these diseases, exceeded the scope of our study. This emphasizes the importance of further research on chronic diseases to explore this relationship more extensively. These limitations should be kept in mind when interpreting the results of the study.

## Conclusions

In conclusion, this study provides new insight into the relationship between the intake of specific nutrients and multimorbidity in the adult population. Investigating the potential impact of specific macro- and micronutrients on the totality of the disease profile of an individual could inform the development of dietary recommendations. The results of the study indicate that individuals with multimorbidity may have higher fat and iron intake, and lower MUFA and Zn intake compared to those with only 0 or 1 chronic condition. These findings suggest that paying attention to specific nutrients, may be an important factor in the prevention and management of multimorbidity. However, further research is needed to confirm these findings and to fully understand the complex relationships between diet, nutrient intake, and the development of multiple chronic conditions.

## Supporting information

Kyprianidou et al. supplementary material 1 Kyprianidou et al. supplementary material

Kyprianidou et al. supplementary material 2 Kyprianidou et al. supplementary material

## Supplementary material

The supplementary material for this article can be found at https://doi.org/10.1017/jns.2023.102
