# Supplementary material for: The association between dietary intake of macro- and micronutrients and multimorbidity: a cross-sectional study in Cyprus
Source: J Nutr Sci. 2023 Nov 24;12:e118. doi: 10.1017/jns.2023.102 (PMC10685255; doi:10.1017/jns.2023.102)
Supplement: Kyprianidou et al. supplementary material 1 — Kyprianidou et al. supplementary material [file S2048679023001027sup001.docx]

**Supplementary File 1**. Non-communicable diseases included in the study according to the International Classification of Diseases (ICD-10).

| **Disease** | **Code** | **Description** |
| --- | --- | --- |
| **Circulatory System** | | |
| **Hyperlipidemia** | E78.5 | Unspecified, a disorder of lipoprotein metabolism other lipidemias. |
| **Hypertension** | I10 | Essential (primary) hypertension, high blood pressure, hypertension (arterial) (benign) (essential) (malignant) (primary) (systemic). |
| **Angina** | I20.9 | Angina pectoris, unspecified. |
| **Atrial fibrillation** | I48.91 | Unspecified atrial fibrillation. |
| **Heart failure** | I50 | Heart failure. |
| **Coronary heart disease** | I25.1 | Atherosclerotic heart diseases of native coronary artery. |
| **Digestive/excretory system** | | |
| **Inflammatory bowel disease/ chronic enteritis/ ulcerative colitis** | K50-K52 | Non infective enteritis and colitis. |
| **Irritating Bowel syndrome** | K58 | Irritable bowel syndrome, irritable colon, spastic colon. |
| **Gastric reflux** | K21 | Gastro-esophageal reflux disease with esophagitis. |
| **Crohn's disease** | K50 | Regional enteritis. |
| **Chronic hepatitis** | K73 | Chronic hepatitis not elsewhere classified. |
| **Cirrhosis** | K74.60 | Unspecified cirrhosis of liver. |
| **Endocrine system** | | |
| **Type 1 diabetes mellitus** | E10 | Type 1 diabetes mellitus. |
| **Type 2 diabetes mellitus** | E11 | Hyperosmolar hyperglycemic state. |
| **Thyroid diseases** | E02, E03.8, E03.9, E05.90, E07.9 | Subclinical iodine-deficiency hypothyroidism; other specified hypothyroidism; hypothyroidism, unspecified; thyrotoxicosis, unspecified without thyrotoxic crisis or storm; disorder of thyroid, unspecified |
| **Polycystic ovarian syndrome** | E28.2 | Polycystic ovarian syndrome, Ovarian dysfunction. |
| **Immune system** | | |
| **Human immunodeficiency virus (HIV)** | B20 | Human immunodeficiency virus [HIV] disease. |
| **Lupus** | M32.9 | Systemic lupus erythematosus, unspecified. |
| **Multiple sclerosis** | G35 | Multiple sclerosis. |
| **Leukemia** | C95.9 | Leukemia, unspecified. |
| **Anemia** | D64.9 |  |
| **Nervous system** | | |
| **Depression** | F33 | Major depressive disorder, recurrent. |
| **Dementia/Alzheimer disease** | G30.9, F03 | Alzheimer's disease, unspecified, Unspecified dementia. |
| **Anorexia/Bulimia** | F50.0, F50.2 | Anorexia nervosa, unspecified, Bulimia nervosa. |
| **Schizophrenia/Bipolar** | F20.9, F31.9 | Schizophrenia unspecified, bipolar disorder, unspecified. |
| **Parkinson disease** | G20 | Parkinson's disease. |
| **Epileptic** | G40.909 | Epilepsy, unspecified, not intractable, without status epilepticus. |
| **Blindness/Low vision** | H54.0 | Blindness, both eyes. |
| **Glaucoma/Cataract** | H40, H25 | Glaucoma, Sentile cataract. |
| **Hearing loss/Deafness** | H90, H91 | Conductive and sensorineural hearing loss, other and unspecified hearing loss. |
| **Renal/urinary system** | | |
| **Chronic kidney disease** | N18.9 | Chronic kidney disease, unspecified. |
| **Reproductive system** | | |
| **Erectile dysfunction** | N52.9 | Unspecified sexual dysfunction not due to a substance or known physiological condition. |
| **Respiratory system** | | |
| **Chronic Bronchitis** | J41, J42 | Simple and mucopurulent chronic bronchitis, Unspecified chronic bronchitis |
| **Chronic Obstructive Pulmonary Disease** | J44.9 | Chronic obstructive pulmonary disease, unspecified |
| **Asthma** | J45 | Asthma. |
| **Chronic sinusitis** | J32 | Chronic sinusitis |
| **Skeletal/muscular system** | | |
| **Rheumatoid arthritis** | MO6.9 | Rheumatoid arthritis, unspecified. |
| **Neoplasms** | | |
| **Colon cancer** | C18.9 | Malignant neoplasm of colon, unspecified. |
| **Melanoma** | C43 | Malignant melanoma of skin. |
| **Urinary cancer** | C67 | Malignant neoplasm of bladder. |
| **Cervical cancer** | C53.9 | Malignant neoplasm of cervix uteri, unspecified. |
| **Ovarian cancer** | C56.9 | Malignant neoplasm of unspecified ovary. |
| **Prostate cancer** | C61 | Malignant neoplasm of prostate. |
| **Breast cancer** | C50.9 | Malignant neoplasm of breast of unspecified site. |
