# Supplementary material for: The association between dietary intake of macro- and micronutrients and multimorbidity: a cross-sectional study in Cyprus
Source: J Nutr Sci. 2023 Nov 24;12:e118. doi: 10.1017/jns.2023.102 (PMC10685255; doi:10.1017/jns.2023.102)
Supplement: Kyprianidou et al. supplementary material 2 — Kyprianidou et al. supplementary material [file S2048679023001027sup002.docx]

**Supplementary Table 1**. Nutritional intake by human systems.

|  | **Energy** | **CHO** | **Sugars** | **Fibres** | **Protein** | **Fat** | **Sat** | **PUFA** | **MUFA** | **Iron** | **Ca** | **Na** | **K** | **Vit. A** | **Vit. E** | **Zinc** |
| --- | --- | --- | --- | --- | --- | --- | --- | --- | --- | --- | --- | --- | --- | --- | --- | --- |
| **Circulatory** | | | | | | | | | | | | | | | | |
| No | 2609.6 ± 8857.7 | 249.4 ± 140.9 | 4.4 ± 8.6 | 23.2 ± 15.9 | 202.7 ± 122.3 | 73.0 ± 46.5 | 29.2 ± 23.2 | 7.1 ± 4.8 | 29.7 ± 19.4 | 25.3 ± 24.8 | 1632.4 ± 1357.0 | 2200.5 ± 1478.5 | 10098.6 ± 10479.5 | 1307.6 ± 2270.6 | 0.5 ± 1.5 | 22.8 ± 71.0 |
| Yes | 2277.3 ± 1613.5 | 261.7 ± 186.8 | 4.3 ± 7.4 | 27.1 ± 24.2 | 187.4 ± 125.2 | 76.9 ± 69.5 | 28.8 ± 27.9 | 7.5 ± 7.0 | 31.4 ± 29.1 | 25.2 ± 24.3 | 1690.8 ± 1462.3 | 2511.5 ± 2556.6 | 12010.1 ± 28835.7 | 1927.6 ± 3648.3 | 0.4 ± 1.0 | 20.2 ± 30.3 |
| *p-value*^*^ | 0.5304 | 0.2439 | 0.7691 | **0.018** | 0.0874 | 0.2872 | 0.7918 | 0.2750 | 0.2482 | 0.9667 | 0.5384 | **0.0123** | 0.1016 | **0.0011** | 0.8192 | 0.5364 |
| **Endocrine** | | | | | | | | | | | | | | | | |
| No | 2567.9 ± 8453.3 | 249.6 ± 144.7 | 4.4 ± 6.7 | 24.2 ± 18.6 | 201.2 ± 122.4 | 72.6 ± 49.6 | 28.9 ± 23.9 | 7.1 ± 5.1 | 29.5 ± 20.7 | 25.6 ± 25.5 | 1640.7 ± 1374.3 | 2217.9 ± 1547.0 | 10407.7 ± 15099.7 | 1442.5 ± 2641.8 | 0.5 ± 1.5 | 23.1 ± 68.9 |
| Yes | 2329.9 ± 1593.8 | 266.3 ± 190.3 | 4.4 ± 13.6 | 24.1 ± 17.5 | 187.7 ± 126.2 | 80.9 ± 67.4 | 30.3 ± 27.0 | 7.8 ± 6.7 | 33.1 ± 28.2 | 23.5 ± 20.4 | 1676.4 ± 1430.2 | 2566.3 ± 2735.4 | 11378.6 ± 24240.2 | 1547.6 ± 2910.0 | 0.4 ± 0.6 | 17.6 ± 22.2 |
| *p-value*^*^ | 0.6947 | 0.1671 | 0.9790 | 0.9200 | 0.1927 | **0.0449** | 0.4526 | 0.0854 | **0.0350** | 0.2828 | 0.7426 | **0.0143** | 0.4676 | 0.6334 | 0.3808 | 0.2693 |
| **Digestive /Excretory** | | | | | | | | | | | | | | | | |
| No | 2582.8 ± 8240.0 | 253.4 ± 149.3 | 4.3 ± 6.6 | 24.4 ± 18.5 | 199.9 ± 123.6 | 74.1 ± 52.3 | 29.2 ± 24.4 | 7.3 ± 5.4 | 30.2 ± 21.9 | 25.1 ± 24.6 | 1657.0 ± 1402.7 | 2274.1 ± 1801.4 | 10609.9 ± 17782.8 | 1422.5 ± 2571.4 | 0.5 ± 1.4 | 22.3 ± 66.6 |
| Yes | 2142.8 ± 1434.8 | 246.4 ± 181.5 | 5.0 ± 15.9 | 22.5 ± 17.9 | 192.3 ± 120.5 | 73.3 ± 59.0 | 28.9 ± 25.1 | 6.9 ± 5.6 | 29.4 ± 24.5 | 26.1 ± 25.4 | 1579.2 ± 1250.0 | 2301.3 ± 1895.0 | 10321.3 ± 10424.3 | 1734.8 ± 3398.6 | 0.4 ± 1.0 | 21.1 ± 34.4 |
| *p-value*^*^ | 0.5243 | 0.6138 | 0.3768 | 0.2386 | 0.5117 | 0.8690 | 0.8878 | 0.5073 | 0.6784 | 0.6423 | 0.5299 | 0.8669 | 0.8498 | 0.2101 | 0.6717 | 0.8288 |
| **Nervous** | | | | | | | | | | | | | | | | |
| No | 2569.5 ± 8147.5 | 252.6 ± 149.6 | 4.4 ± 8.5 | 24.1 ± 18.3 | 202.1 ± 124.4 | 74.3 ± 52.7 | 29.5 ± 24.8 | 7.2 ± 5.4 | 30.2 ± 22.0 | 25.4 ± 24.5 | 1649.7 ± 1356.3 | 2274.4 ± 1804.4 | 10644.8 ± 17637.5 | 1430.3 ± 2515.0 | 0.4 ± 1.4 | 22.6 ± 66.0 |
| Yes | 2170.4 ± 1529.2 | 251.8 ± 185.9 | 4.3 ± 6.7 | 24.9 ± 19.5 | 174.0 ± 109.8 | 71.7 ± 57.6 | 26.1 ± 21.4 | 7.1 ± 5.6 | 29.0 ± 24.3 | 23.3 ± 25.0 | 1608.1 ± 1601.3 | 2303.9 ± 1893.0 | 9983.4 ± 10593.1 | 1713.1 ± 3895.8 | 0.4 ± 1.0 | 16.7 ± 29.1 |
| *p-value*^*^ | 0.5941 | 0.9559 | 0.8724 | 0.6384 | 0.0233 | 0.6134 | 0.1573 | 0.7712 | 0.5754 | 0.3785 | 0.7553 | 0.8671 | 0.6888 | 0.2964 | 0.9229 | 0.3333 |
| **Respiratory** | | | | | | | | | | | | | | | | |
| No | 2563.9 ± 8084.5 | 253.0 ± 147.8 | 4.3 ± 8.5 | 24.3 ± 18.3 | 201.3 ± 123.5 | 73.9 ± 52.0 | 29.2 ± 24.3 | 7.2 ± 5.4 | 30.2 ± 21.7 | 24.4 ± 24.7 | 1658.1 ± 1384.5 | 2269.5 ± 1779.7 | 10766.1 ± 17615.6 | 1476.3 ± 2675.3 | 0.5 ± 1.4 | 22.5 ± 65.8 |
| Yes | 2165.2 ± 1615.3 | 248.3 ± 204.0 | 5.0 ± 7.0 | 23.4 ± 19.1 | 173.9 ± 116.2 | 74.7 ± 63.9 | 28.6 ± 26.0 | 7.0 ± 5.8 | 23.4 ± 26.9 | 23.4 ± 24.1 | 1543.3 ± 1380.8 | 2355.2 ± 2118.7 | 8734.8 ± 9233.8 | 1276.0 ± 2812.7 | 0.4 ± 1.0 | 18.7 ± 30.7 |
| *p-value** | 0.6160 | 0.7672 | 0.4475 | 0.6367 | **0.0377** | 0.8927 | 0.8142 | 0.6738 | 0.7378 | 0.4274 | 0.4200 | 0.6462 | 0.2464 | 0.4803 | 0.6606 | 0.5606 |
| **Immune** | | | | | | | | | | | | | | | | |
| No | 2539.8 ± 7839.2 | 252.5 ± 154.0 | 4.4 ± 8.4 | 24.3 ± 18.6 | 198.7 ± 122.8 | 74.2 ± 52.6 | 29.2 ± 24.2 | 7.2 ± 5.4 | 30.2 ± 22.1 | 25.2 ± 24.8 | 1644.3 ± 1380.4 | 2292.4 ± 1827.2 | 10521.1 ± 17172.5 | 1465.2 ± 2714.2 | 0.4 ± 1.4 | 22.3 ± 64.3 |
| Yes | 2154.7 ± 1339.7 | 254.6 ± 145.5 | 4.2 ± 6.0 | 21.8 ± 12.6 | 207.0 ± 134.7 | 68.5 ± 71.4 | 27.6 ± 32.5 | 6.3 ± 6.0 | 25.7 ± 27.6 | 26.5 ± 20.0 | 1761.5 ± 1502.8 | 1868.3 ± 1193.6 | 12503.4 ± 11543.4 | 1349.3 ± 1751.1 | 0.6 ± 1.8 | 18.0 ± 24.5 |
| *p-value*^*^ | 0.7716 | 0.9374 | 0.8930 | 0.4266 | 0.6996 | 0.5335 | 0.7016 | 0.3057 | 0.2394 | 0.7648 | 0.6220 | 0.1730 | 0.4980 | 0.8049 | 0.4109 | 0.6904 |
| **Skeletal/Muscular** | | | | | | | | | | | | | | | | |
| No | 2529.9 ± 7824.9 | 251.9 ± 153.7 | 4.3 ± 6.6 | 24.2 ± 18.4 | 199.2 ± 122.3 | 73.7 ± 52.6 | 29.1 ± 24.6 | 7.2 ± 5.3 | 29.9 ± 21.9 | 25.2 ± 24.7 | 1645.0 ± 1382.5 | 2262.7 ± 1790.8 | 10539.2 ± 17172.6 | 1446.9 ± 2652.8 | 0.5 ± 1.4 | 22.3 ± 64.1 |
| Yes | 2420.7 ± 1487.5 | 271.4 ± 150.3 | 7.6 ± 31.6 | 24.0 ± 19.5 | 189.7 ± 151.4 | 83.8 ± 71.4 | 28.2 ± 20.6 | 8.7 ± 8.1 | 35.6 ± 32.8 | 26.0 ± 23.7 | 1712.4 ± 1439.1 | 2805.0 ± 2407.2 | 11804.0 ± 10630.9 | 1920.0 ± 3724.0 | 0.4 ± 0.9 | 18.7 ± 25.1 |
| *p-value*^*^ | 0.9372 | 0.4805 | **0.0272** | 0.9563 | 0.6777 | 0.2920 | 0.8266 | 0.1144 | 0.1591 | 0.8678 | 0.7861 | 0.0951 | 0.6788 | 0.3429 | 0.9349 | 0.7520 |
| **Neoplasm** | | | | | | | | | | | | | | | | |
| No | 2531.1 ± 7793.4 | 252.1 ± 153.1 | 4.4 ± 8.4 | 24.1 ± 18.3 | 199.2 ± 123.1 | 74.1 ± 52.9 | 29.1 ± 24.4 | 7.2 ± 5.4 | 30.2 ± 22.1 | 25.1 ± 24.5 | 1638.2 ± 1364.0 | 2273.0 ± 1806.4 | 10536.0 ± 17111.5 | 1463.2 ± 2705.2 | 0.4 ± 1.4 | 22.0 ± 63.7 |
| Yes | 2312.5 ± 1735.9 | 274.4 ± 184.2 | 5.2 ± 6.4 | 27.6 ± 21.7 | 187.2 ± 127.9 | 69.0 ± 68.0 | 28.1 ± 27.2 | 6.3 ± 6.2 | 27.2 ± 28.1 | 30.1 ± 28.1 | 1994.8 ± 2144.0 | 2559.9 ± 2131.2 | 12233.8 ± 12186.8 | 1356.0 ± 1627.8 | 0.4 ± 0.6 | 20.2 ± 25.8 |
| *p-value*^†^ | 0.5680 | 0.643 | 0.247 | 0.448 | 0.628 | 0.117 | 0.862 | 0.261 | 0.070 | 0.357 | 0.910 | 0.691 | 0.916 | 0.248 | 0.640 | 0.658 |
| **Renal/Urinary** | | | | | | | | | | | | | | | | |
| No | 2534.5 ± 7754.6 | 252.8 ± 154.1 | 4.4 ± 8.4 | 24.2 ± 18.5 | 199.6 ± 123.0 | 74.2 ± 5.4 | 29.2 ± 24.5 | 7.2 ± 5.4 | 30.2 ± 22.3 | 25.3 ± 24.7 | 1650.5 ± 1385.5 | 2289.8 ± 1816.0 | 10573.0 ± 17081.2 | 1466.3 ± 2698.0 | 0.5 ± 1.4 | 22.3 ± 63.7 |
| Yes | 1743.7 ± 839.5 | 220.1 ± 97.0 | 2.2 ± 2.6 | 20.6 ± 10.7 | 140.4 ± 127.7 | 49.8 ± 34.9 | 19.9 ± 18.7 | 4.8 ± 3.9 | 21.2 ± 15.3 | 19.0 ± 16.6 | 1274.6 ± 1162.8 | 1065.3 ± 538.0 | 10760.0 ± 9363.6 | 861.5 ± 1245.3 | 0.1 ± 0.1 | 8.5 ± 8.0 |
| *p-value*^†^ | 0.083 | 0.632 | 0.518 | 0.534 | 0.126 | **0.033** | **0.018** | 0.093 | 0.214 | 0.281 | 0.477 | **0.001** | 0.530 | 0.278 | 0.182 | **0.029** |
| **Reproductive** | | | | | | | | | | | | | | | | |
| No | 2529.7 ± 7751.3 | 252.7 ± 153.8 | 4.4 ± 8.3 | 24.2 ± 18.5 | 199.2 ± 123.4 | 73.9 ± 53.2 | 29.1 ± 24.5 | 7.2 ± 5.4 | 30.1 ± 22.3 | 25.3 ± 24.6 | 1649.2 ± 1386.9 | 2279.7 ± 1817.1 | 10604.2 ± 17087.1 | 1463.6 ± 2696.7 | 0.4 ± 1.4 | 22.1 ± 63.4 |
| Yes | 2171.7 ± 1145.5 | 225.2 ± 138.3 | 5.6 ± 11.6 | 21.6 ± 10.8 | 164.8 ±  85.9 | 84.9 ± 57.2 | 28.7 ± 19.2 | 8.0 ± 5.1 | 34.1 ± 20.9 | 14.0 ± 5.7 | 1123.9 ± 457.6 | 2132.2 ± 1166.3 | 6150.1 ± 4110.7 | 1138.0 ± 1206.7 | 0.2 ± 0.4 | 9.2 ± 5.5 |
| *p-value*^†^ | 0.951 | 0.590 | 0.877 | 0.782 | 0.796 | 0.468 | 0.988 | 0.340 | 0.402 | 0.159 | 0.247 | 0.960 | 0.512 | 0.867 | 0.923 | 0.488 |
| Abbreviations: CHO: carbohydrates; Sat: saturated; PUFA: poly-unsaturated fat; MUFA: mono-unsaturated fat; Ca: calcium; Na: sodium; K: potassium; Vit. A: vitamin A; Vit E.: vitamin E; *Differences between groups were tested using t-test; ^†^Differences between groups were tested using Kolmogorov-Smirnov test; Data are presented as mean ± standard deviation; Bold values indicate statistically significant associations (p<0.05). | | | | | | | | | | | | | | | | |

**Supplementary Table 2**. Nutritional intake by sociodemographic (age group categories, sex, geographical area, and residency) and by socioeconomics (marital status, educational level, annual income, and job status) characteristics.

|  | **Energy** | **CHO** | **Sugars** | **Fibres** | **Protein** | **Fat** | **Sat** | **PUFA** | **MUFA** | **Iron** | **Ca** | **Na** | **K** | **Vit. A** | **Vit. E** | **Zinc** |
| --- | --- | --- | --- | --- | --- | --- | --- | --- | --- | --- | --- | --- | --- | --- | --- | --- |
| **Age group** | | | | | | | | | | | | | | | | |
| 18-24 | 2368.5 ± 1216.2 | 271.3 ± 151.7 | 6.7 ± 16.2 | 22.5 ± 16.7 | 214.6 ± 121.6 | 76.9 ± 44.1 | 35.9 ± 31.4 | 7.5 ± 4.8 | 30.2 ± 17.6 | 27.1 ± 28.7 | 1779.5 ± 1492.6 | 2373.2 ± 1715.0 | 9972.8 ± 10533.5 | 1070.5 ± 1790.9 | 0.6 ± 2.8 | 34.7 ± 146.7 |
| 25-44 | 2566.5 ± 6985.6 | 258.6 ± 169.5 | 4.4 ± 5.7 | 24.4 ± 19.0 | 210.8 ± 124.1 | 77.1 ± 56.0 | 30.3 ± 24.5 | 7.5 ± 5.7 | 31.1 ± 23.1 | 25.8 ± 24.3 | 1644.4 ± 1351.8 | 2354.9 ± 2037.1 | 10002.0 ± 10251.9 | 1426.3 ± 2643.6 | 0.4 ± 1.0 | 22.1 ± 33.6 |
| 45-64 | 2046.4 ± 1119.3 | 235.5 ± 130.8 | 3.9 ± 6.7 | 24.5 ± 19.2 | 184.1 ± 120.9 | 67.6 ± 48.7 | 24.8 ± 19.0 | 6.7 ± 5.1 | 28.0 ± 21.5 | 24.3 ± 24.1 | 1563.0 ± 1250.7 | 2122.5 ± 1432.0 | 10940.0 ± 21616.6 | 1604.7 ± 3123.3 | 0.4 ± 1.0 | 18.9 ± 30.9 |
| 65+ | 3692.0 ± 17618.4 | 244.9 ± 136.5 | 2.9 ± 4.8 | 24.7 ± 16.2 | 170.0 ± 118.7 | 73.3 ± 61.0 | 25.9 ± 23.6 | 7.1 ± 5.6 | 30.7± 25.4 | 22.9 ± 21.9 | 1686.9 ± 1638.9 | 2222.8 ± 1784.4 | 12703.7 ± 28624.7 | 1744.5 ± 2635.5 | 0.3 ± 0.6 | 14.5 ± 21.7 |
| *p-value*^*^ | 0.2260 | 0.0581 | **0.0003** | 0.6342 | **0.0005** | 0.0784 | **<0.001** | 0.2262 | 0.2604 | 0.4111 | 0.4226 | 0.2805 | 0.3858 | 0.1341 | 0.2201 | **0.0248** |
| **Sex** | | | | | | | | | | | | | | | | |
| Males | 2237.9 ± 1284.0 | 252.8 ± 154.3 | 3.8 ± 6.1 | 24.4 ± 20.0 | 202.5 ± 124.6 | 75.5 ± 54.8 | 29.9 ± 26.6 | 7.4 ± 5.3 | 30.7 ± 22.7 | 24.8 ± 23.9 | 1644.3 ± 1390.5 | 2326.9 ± 1752.4 | 9824.8 ± 10229.8 | 1472.2 ± 2781.9 | 0.5 ± 1.7 | 23.2 ± 87.3 |
| Females | 2749.9 ± 10210.0 | 252.2 ± 153.2 | 4.8 ± 9.7 | 24.0 ± 17.1 | 196.1 ± 122.3 | 72.9 ± 51.9 | 28.5 ± 22.7 | 7.1 ± 5.5 | 29.6 ± 21.9 | 25.6 ± 25.3 | 1648.9 ± 1379.2 | 2240.2 ± 1857.0 | 11154.0 ± 20790.4 | 1450.8 ± 2614.7 | 0.4 ± 1.0 | 21.4 ± 35.1 |
| *p-value*^†^ | 0.2677 | 0.9527 | **0.0401** | 0.7484 | 0.4082 | 0.4091 | 0.3176 | 0.2850 | 0.3901 | 0.6156 | 0.9559 | 0.4242 | 0.1920 | 0.8971 | 0.7950 | 0.6292 |
| **Geographical area** | | | | | | | | | | | | | | | | |
| Nicosia | 2637.3 ± 9282.9 | 252.8 ± 166.7 | 3.7 ± 5.5 | 23.2± 18.7 | 191.1 ± 121.1 | 75.4 ± 59.1 | 28.8 ± 24.3 | 7.3 ± 6.1 | 30.8 ± 24.6 | 23.1 ± 21.5 | 1632.3 ± 1465.9 | 2293.2 ± 2106.1 | 9815.1 ± 10228.8 | 1391.2 ± 2451.6 | 0.4 ± 0.8 | 17.5 ± 25.8 |
| Limassol | 2613.9 ± 8974.6 | 238.8 ± 141.4 | 5.5 ± 8.3 | 23.9 ± 18.2 | 186.2 ± 115.3 | 74.1 ± 48.6 | 29.3 ± 24.4 | 7.3 ± 4.8 | 30.5 ± 20.9 | 22.4 ± 21.5 | 1406.4 ± 1037.7 | 2097.8 ± 1442.4 | 8674.9 ± 9950.1 | 2016.2 ± 3550.0 | 0.6 ± 1.4 | 24.1 ± 108.4 |
| Larnaca | 2091.9 ± 1086.8 | 247.1 ± 130.8 | 5.1 ± 14.8 | 25.6 ± 19.4 | 210.6 ± 130.0 | 68.7 ± 49.2 | 26.4 ± 23.5 | 6.7 ± 4.4 | 27.7 ± 19.9 | 25.2 ± 26.6 | 1609.6 ± 1260.1 | 2224.7 ± 1332.8 | 11242.4 ± 25635.4 | 1100.1 ± 2060.8 | 0.6 ± 2.7 | 21.0 ± 36.0 |
| Paphos | 2405.4 ± 1111.9 | 267.4 ± 138.2 | 3.6 ± 5.6 | 24.5 ± 14.4 | 228.5 ± 133.9 | 74.6 ± 42.6 | 33.0 ± 25.0 | 7.3 ± 4.8 | 29.6 ± 18.0 | 37.7 ± 33.0 | 2059.7 ± 1418.6 | 2537.6 ± 1860.5 | 16652.2 ± 33162.5 | 1107.4 ± 1849.5 | 0.3 ± 0.5 | 34.6 ± 46.4 |
| Ammochostos | 2674.8 ± 1487.7 | 319.4 ± 182.1 | 3.6 ± 4.6 | 29.4 ± 20.7 | 250.4 ± 120.7 | 75.9 ± 55.1 | 31.5 ± 27.3 | 7.8 ± 6.4 | 29.7 ± 22.4 | 36.4 ± 32.4 | 2495.1 ± 2105.2 | 2846.5 ± 1945.4 | 13933.8 ± 10888.9 | 639.9 ± 911.6 | 0.3 ± 0.4 | 32.4 ± 46.6 |
| *p-value*^*^ | 0.9480 | **0.0100** | **0.0192** | 0.1641 | **0.0006** | 0.7092 | 0.2332 | 0.7221 | 0.6250 | **<0.001** | **<0.001** | **0.0328** | **0.0002** | **0.0002** | 0.1742 | 0.0675 |
| **Residency** | | | | | | | | | | | | | | | | |
| Urban | 2563.4 ± 8827.8 | 243.8 ± 145.9 | 4.5 ± 6.8 | 23.8 ± 18.0 | 191.9 ± 119.9 | 72.5 ± 50.3 | 28.5 ± 23.2 | 7.1 ± 5.1 | 29.5 ± 21.2 | 24.6 ± 24.4 | 1572.1 ± 1270.2 | 2163.4 ± 1621.3 | 9947.7 ± 15270.3 | 1542.4 ± 2759.6 | 0.5 ± 1.5 | 23.0 ± 70.8 |
| Rural | 2420.3 ± 1461.6 | 280.5 ± 174.3 | 4.2 ± 12.0 | 25.3 ± 19.8 | 223.9 ± 131.3 | 78.9 ± 61.4 | 31.1 ± 27.9 | 7.7 ± 6.3 | 32.1 ± 25.3 | 27.3 ± 25.7 | 1895.9 ± 1684.1 | 2650.5 ± 2293.1 | 12608.2 ± 21706.3 | 1208.5 ± 2453.4 | 0.3 ± 0.5 | 19.9 ± 29.8 |
| *p-value*^†^ | 0.7913 | **0.0006** | 0.6834 | 0.2586 | **0.0005** | 0.0820 | 0.1248 | 0.1219 | 0.0941 | 0.1210 | **0.0008** | **0.0001** | **0.0254** | 0.0859 | 0.1182 | 0.4836 |
| **Marital status** | | | | | | | | | | | | | | | | |
| Married | 2188.5 ± 1256.5 | 250.7 ± 147.7 | 4.0 ± 6.3 | 24.8 ± 18.7 | 194.5 ± 121.4 | 72.9 ± 55.5 | 27.2 ± 21.4 | 7.1 ± 5.7 | 30.0 ± 23.2 | 25.3 ± 23.4 | 1684.9 ± 1347.8 | 2284.6 ± 1895.7 | 10972.5 ± 16338.7 | 1517.9 ± 2855.8 | 0.4 ± 0.9 | 19.5 ± 29.8 |
| Unmarried | 2619.2 ± 7749.6 | 253.7 ± 164.2 | 5.1 ± 10.9 | 23.0 ± 18.3 | 209.7 ± 125.1 | 76.3 ± 49.8 | 32.9 ± 28.8 | 7.4 ± 5.0 | 30.4 ± 20.4 | 25.1 ± 26.2 | 1588.4 ± 1413.6 | 2210.5 ± 1497.5 | 9991.4 ± 19163.5 | 1260.3 ± 2239.2 | 0.5 ± 1.9 | 26.7 ± 96.7 |
| Divorced  /Widowed | 4312.2 ± 20796.3 | 260.0 ± 146.9 | 3.8 ± 6.4 | 25.3 ± 17.3 | 175.9 ± 118.8 | 71.0 ± 53.9 | 24.8 ± 20.0 | 7.1 ± 6.1 | 29.3 ± 23.9 | 25.2 ± 25.5 | 1658.5 ± 1473.9 | 2432.5 ± 2154.8 | 10813.4 ± 10665.9 | 2032.8 ± 3328.5 | 0.5 ± 1.4 | 18.4 ± 27.7 |
| *p-value*^*^ | **0.0418** | 0.8457 | 0.1052 | 0.2470 | **0.0338** | 0.5163 | **0.0003** | 0.6999 | 0.9035 | 0.9889 | 0.5430 | 0.5207 | 0.6567 | **0.0393** | 0.3803 | 0.1686 |
| **Educational level** | | | | | | | | | | | | | | | | |
| Primary education | 2267.7 ± 1389.3 | 254.5 ± 142.5 | 2.7 ± 4.3 | 26.7 ± 17.2 | 183.6 ± 117.7 | 74.9 ± 63.5 | 27.3 ± 27.8 | 7.1 ± 5.2 | 31.5 ± 26.5 | 25.9 ± 24.5 | 1806.5 ± 1828.6 | 2409.7 ± 1700.6 | 16464.5 ± 39443.8 | 1922.9 ± 2783.0 | 0.3 ± 0.6 | 15.0 ± 23.7 |
| Secondary education | 2224.2 ± 1305.1 | 257.1 ± 155.2 | 4.4 ± 11.4 | 23.8 ± 19.0 | 196.5 ± 130.2 | 71.9 ± 50.3 | 28.2 ± 23.7 | 7.1 ± 5.5 | 29.4 ± 21.2 | 26.1 ± 26.2 | 1700.4 ± 1520.4 | 2347.4 ± 2045.7 | 10777.5 ± 11064.7 | 1438.2 ± 2555.0 | 0.5 ± 2.1 | 25.4 ± 104.3 |
| Higher education | 2698.1 ± 9595.2 | 250.7 ± 154.3 | 4.6 ± 6.8 | 24.2 ± 18.3 | 201.7 ± 120.0 | 75.0 ± 53.6 | 29.8 ± 24.6 | 7.3 ± 5.5 | 30.3 ± 22.3 | 24.9 ± 24.0 | 1614.4 ± 1269.0 | 2236.7 ± 1709.0 | 9971.1 ± 15888.3 | 1435.9 ± 2748.5 | 0.5 ± 1.0 | 21.4 ± 34.4 |
| *p-value*^*^ | 0.6238 | 0.8193 | 0.2321 | 0.5031 | 0.5054 | 0.6710 | 0.5163 | 0.9086 | 0.7339 | 0.7304 | 0.4132 | 0.5447 | **0.0119** | 0.4157 | 0.7749 | 0.4060 |
| **Annual income** | | | | | | | | | | | | | | | | |
| Low | 2818.7 ± 10159.8 | 244.1 ± 139.4 | 5.3 ± 13.3 | 22.6 ± 15.3 | 196.0 ± 124.6 | 73.0 ± 47.9 | 31.6 ± 30.0 | 7.3 ± 4.9 | 29.7 ± 19.5 | 24.9 ± 24.9 | 1552.5 ± 1361.6 | 2130.6 ± 1571.2 | 11007.4 ± 22406.3 | 1322.5 ± 2102.2 | 0.6 ± 2.2 | 28.0 ± 122.4 |
| Middle | 2606.3 ± 8683.7 | 257.9 ± 151.6 | 4.4 ± 6.8 | 24.8 ± 19.5 | 207.3 ± 123.3 | 75.4 ± 52.8 | 29.5 ± 23.4 | 7.4 ± 5.5 | 30.5 ± 22.2 | 25.5 ± 25.2 | 1651.5 ± 1392.8 | 2288.5 ± 1679.7 | 10207.9 ± 10611.5 | 1520.3 ± 2802.2 | 0.5 ± 1.2 | 20.4 ± 32.9 |
| High | 2182.6 ± 1368.2 | 248.9 ± 167.4 | 3.7 ± 5.6 | 24.2 ± 18.5 | 186.1 ± 120.6 | 72.5 ± 57.7 | 26.8 ± 21.6 | 6.9 ± 5.7 | 29.6 ± 24.1 | 25.2 ± 23.8 | 1698.7 ± 1386.9 | 2363.6 ± 2174.4 | 10988.2 ± 21084.7 | 1450.4 ± 2868.5 | 0.4 ± 0.7 | 21.2 ± 32.5 |
| *p-value*^*^ | 0.5929 | 0.4520 | 0.0747 | 0.3042 | 0.0599 | 0.7058 | 0.0654 | 0.5453 | 0.8031 | 0.9393 | 0.4542 | 0.3120 | 0.7406 | 0.6528 | 0.2923 | 0.2762 |
| **Job status** | | | | | | | | | | | | | | | | |
| Private employee | 2667.9 ± 7678.9 | 263.2 ± 172.4 | 4.5 ± 6.7 | 24.7 ± 19.9 | 215.7 ± 127.8 | 78.1 ± 55.2 | 31.0 ± 24.7 | 7.5 ± 5.5 | 31.5 ± 23.2 | 26.5 ± 24.9 | 1627.0 ± 1222.5 | 2306.4 ± 1652.3 | 11029.0 ± 19255.5 | 1691.6 ± 3326.2 | 0.4 ± 1.1 | 21.9 ± 33.2 |
| State  employee | 2079.1 ± 1189.7 | 246.3 ± 143.5 | 3.6 ± 6.1 | 24.1 ± 18.5 | 174.9 ± 109.7 | 65.9 ± 49.9 | 24.4 ± 17.2 | 6.4 ± 6.0 | 27.1 ± 22.1 | 24.4 ± 23.3 | 1714.2 ± 1401.8 | 2317.7 ± 2286.3 | 10547.9 ± 10613.9 | 1297.5 ± 2310.6 | 0.4 ± 0.9 | 18.9 ± 31.0 |
| Freelance | 2095.0 ± 1193.3 | 235.8 ± 133.6 | 4.4 ± 6.5 | 24.1 ± 17.3 | 206.2 ± 120.6 | 70.0 ± 52.3 | 26.8 ± 24.0 | 7.2 ± 5.2 | 28.6 ± 20.8 | 25.9 ± 27.0 | 1625.6 ± 1435.3 | 2207.3 ± 1433.4 | 9248.3 ± 9960.3 | 928.5 ± 1403.8 | 0.5 ± 1.4 | 23.0 ± 41.6 |
| Unemployed | 2205.7 ± 1234.9 | 245.5 ± 146.7 | 4.7 ± 6.9 | 22.3 ± 15.6 | 197.4 ± 125.8 | 75.2 ± 47.6 | 33.2 ± 31.0 | 7.4 ± 4.7 | 30.6 ± 19.5 | 24.8 ± 26.1 | 1600.1 ± 1519.3 | 2206.9 ± 1873.2 | 9389.0 ± 10328.1 | 1207.5 ± 1863.6 | 0.6 ± 2.5 | 21.8 ± 34.0 |
| Retired | 3795.4 ± 17952.3 | 249.1 ± 138.8 | 3.3 ± 5.8 | 25.4 ± 17.3 | 172.8 ± 120.9 | 75.5 ± 62.6 | 26.4 ± 24.0 | 7.4 ± 5.9 | 31.7 ± 26.3 | 23.0 ± 22.3 | 1733.8 ± 1672.0 | 2293.2 ± 1798.6 | 12681.8 ± 29144.5 | 1839.2 ± 2917.5 | 0.4 ± 0.7 | 14.9 ± 22.1 |
| *p-value*^*^ | 0.3103 | 0.3987 | 0.1530 | 0.5319 | **0.0004** | 0.0804 | **0.0009** | 0.2028 | 0.1644 | 0.6212 | 0.8560 | 0.9553 | 0.4453 | **0.0229** | 0.7189 | 0.1909 |
| Abbreviations: CHO: carbohydrates; Sat: saturated; PUFA: poly-unsaturated fat; MUFA: mono-unsaturated fat; Ca: calcium; Na: sodium; K: potassium; Vit. A: vitamin A; Vit E.: vitamin E; ^*^Differences between groups were tested using ANOVA test; ^†^Differences between groups were tested using t-test; Data are presented as mean ± standard deviation; Bold values indicate statistically significant associations (p<0.05). | | | | | | | | | | | | | | | | |

**Supplementary Table 3**. Nutritional intake by lifestyle characteristics (smoking status, physical activity level, Mediterranean Diet adherence, quality of sleep and BMI category).

|  | **Energy** | **CHO** | **Sugars** | **Fibres** | **Protein** | **Fat** | **Sat** | **PUFA** | **MUFA** | **Iron** | **Ca** | **Na** | **K** | **Vit. A** | **Vit. E** | **Zinc** |
| --- | --- | --- | --- | --- | --- | --- | --- | --- | --- | --- | --- | --- | --- | --- | --- | --- |
| **Smoking status** | | | | | | | | | | | | | | | | |
| Non-smoker | 2746.4 ± 9604.4 | 254.4 ± 160.6 | 4.8 ± 9.4 | 24.3 ± 18.8 | 198.4 ± 120.5 | 77.3 ± 57.1 | 31.1 ± 27.3 | 7.5 ± 5.7 | 31.4 ± 24.0 | 24.5 ± 23.5 | 1621.0 ± 1353.8 | 2258.4 ± 1798.7 | 10406.4 ± 19720 | 1402.8 ± 2660.6 | 0.5 ± 1.6 | 22.8 ± 74.5 |
| Current smoker | 2131.4 ± 248.2 | 248.2 ± 142.5 | 3.7 ± 6.0 | 24.1 ± 17.8 | 199.1 ± 127.3 | 68.0 ± 45.0 | 25.6 ± 18.0 | 6.8 ± 5.0 | 27.7 ± 18.6 | 26.5 ± 26.6 | 1689.6 ± 1443.9 | 2306.5 ± 1849.7 | 10815.5 ± 10654.0 | 1560.2 ± 2750.3 | 0.3 ± 0.8 | 21.2 ± 36.1 |
| *p-value*^*^ | 0.2014 | 0.5171 | **0.0311** | 0.9049 | 0.9367 | **0.0049** | **0.004** | **0.0453** | 0.069 | 0.2045 | 0.4264 | 0.6703 | 0.6996 | 0.6321 | **0.0338** | 0.6946 |
| **Physical activity level** | | | | | | | | | | | | | | | | |
| Physical inactive | 2822.5 ± 10651.5 | 254.0 ± 167.7 | 4.3 ± 9.5 | 24.4 ± 19.4 | 196.4 ± 126.9 | 75.3 ± 59.2 | 28.3 ± 24.4 | 7.2± 5.5 | 30.7 ± 24.4 | 24.1 ± 23.4 | 1649.1 ± 1464.2 | 2285.1 ± 1735.9 | 11073.2 ± 21416.4 | 1536.6 ± 3073.5 | 0.4 ± 1.7 | 17.7 ± 27.9 |
| Physical active | 2206.9 ± 1117.0 | 250.9 ± 137.6 | 4.5 ± 6.9 | 24.0 ± 17.3 | 201.1 ± 118.4 | 72.6 ± 45.9 | 30.1 ± 24.7 | 7.2 ± 5.4 | 29.4 ± 19.6 | 26.4 ± 26.0 | 1644.3 ± 1298.4 | 2268.7 ± 1902.9 | 9958.9 ± 10323.0 | 1384.9 ± 2204.7 | 0.5 ± 1.0 | 27.3 ± 87.1 |
| *p-value*^*^ | 0.1825 | 0.7292 | 0.7792 | 0.7534 | 0.5485 | 0.4104 | 0.2215 | 0.9763 | 0.3301 | 0.1072 | 0.9540 | 0.8800 | 0.2732 | 0.3598 | 0.5990 | 0.0115 |
| **Mediterranean Diet adherence** | | | | | | | | | | | | | | | | |
| Low (≤13) | 2496.9 ± 10527.3 | 226.6 ± 146.7 | 4.1 ± 11.0 | 22.1 ± 17.9 | 179.0 ± 120.8 | 65.8 ± 48.8 | 25.8 ± 22.5 | 6.4 ± 5.1 | 26.9 ± 20.2 | 22.4 ± 22.5 | 1428.6 ± 1201.9 | 2013.1 ± 1939.3 | 9915.3 ± 19893.9 | 1558.2 ± 3052.2 | 0.4 ± 1.9 | 18.1 ± 30.8 |
| Moderate (14-17) | 2528.7 ± 7817.0 | 244.5 ± 149.6 | 4.0 ± 5.8 | 22.7 ± 16.5 | 199.2 ± 123.1 | 71.7± 53.4 | 27.9 ± 23.5 | 6.9 ± 5.3 | 29.1 ± 22.4 | 24.5 ± 23.5 | 1620.6 ± 1337.2 | 2191.7 ± 1452.4 | 10436.4 ± 10642.4 | 1375.0 ± 2453.2 | 0.4 ± 1.0 | 19.8 ± 32.2 |
| High (≥18) | 2557.4 ± 1317.5 | 290.5 ± 158.8 | 5.2 ± 7.5 | 28.3 ± 20.4 | 222.3 ± 122.2 | 85.8 ± 55.5 | 34.3 ± 26.8 | 8.5 ± 5.7 | 34.8 ± 23.4 | 29.2 ± 27.7 | 1918.1 ± 1569.9 | 2672.0 ± 1984.1 | 11464.7 ± 19605.7 | 1453.7 ± 2530.7 | 0.5± 1.0 | 29.54.1 ± 104.3 |
| *p-value*^†^ | 0.9945 | **<0.001** | 0.1327 | **<0.001** | **<0.001** | **<0.001** | **<0.001** | **<0.001** | **<0.001** | **0.0007** | **<0.001** | **<0.001** | 0.4636 | 0.6516 | 0.6804 | 0.0347 |
| **Quality of sleep** | | | | | | | | | | | | | | | |  |
| Good (≤ 5) | 2519.5 ± 7846.5 | 255.4 ± 161.4 | 4.3 ± 6.6 | 24.8 ± 18.6 | 198.9 ± 120.7 | 73.6 ± 53.3 | 28.8 ± 23.9 | 7.2 ± 5.6 | 30.0 ± 22.4 | 26.2 ± 25.8 | 1680.2 ± 1442.5 | 2302.3 ± 1941.4 | 11459.0 ± 20338.1 | 1459.0 ± 2741.2 | 0.5 ± 1.6 | 21.0 ± 34.6 |
| Poor (>5) | 2538.2 ± 7522.1 | 248.0 ± 1408.8 | 4.5 ± 10.4 | 23.2 ± 18.0 | 199.0 ± 127.1 | 74.6 ± 53.1 | 29.6 ± 25.3 | 7.3 ± 5.2 | 30.2 ± 22.0 | 23.7 ± 22.7 | 1595.2 ± 1286.5 | 2240.3 ± 1591.4 | 9201.8 ± 9730.0 | 1461.9 ± 2605.4 | 0.4 ± 0.9 | 24.0 ± 91.7 |
| *p-value*^*^ | 0.9682 | 0.4313 | 0.7655 | 0.1332 | 0.9888 | 0.7778 | 0.5846 | 0.7876 | 0.9253 | 0.0894 | 0.3127 | 0.5740 | **0.0291** | 0.9860 | 0.2864 | 0.4236 |
| **BMI** | | | | | | | | | | | | | | | | |
| Normal | 2377.7 ± 6687.5 | 241.3 ± 135.6 | 4.8 ± 9.9 | 23.2 ± 16.0 | 197.7± 124.0 | 70.0 ± 45.4 | 27.6± 20.8 | 6.8 ± 5.1 | 28.3 ± 19.0 | 24.5 ± 24.1 | 1577.6 ± 1316.3 | 2150.6 ± 1828.1 | 10288.3± 17362.2 | 1443.6 ± 2471.1 | 0.5 ± 1.7 | 23.5 ± 83.4 |
| Underweight | 2261.0 ± 1061.0 | 241.0 ± 119.7 | 5.7 ± 8.2 | 22.7 ± 12.9 | 204.9 ± 118.2 | 86.2 ± 43.3 | 34.5 ± 20.6 | 8.3 ± 4.5 | 34.3 ± 16.8 | 21.5 ± 22.7 | 1444.9 ± 1155.2 | 2176.8 ± 1234.6 | 7274.6 ± 8603.7 | 1128.5 ± 1702.7 | 0.4 ± 0.5 | 17.5 ± 31.5 |
| Overweight | 2919.6 ± 10804.9 | 269.1 ± 174.3 | 3.9 ± 6.4 | 25.8 ± 22.2 | 202.5 ± 119.0 | 78.1 ± 60.8 | 31.2 ± 29.1 | 7.6 ± 5.9 | 31.5 ± 24.8 | 26.8 ± 25.3 | 1791.9 ± 1444.3 | 1791.9 ± 1788.5 | 2472.7 ± 10786.4 | 1441.2± 2658.1 | 0.4 ± 1.1 | 21.9 ± 34.0 |
| Obese | 2206.9 ± 1419.8 | 250.7 ± 169.6 | 3.7 ± 5.5 | 24.1 ± 18.8 | 188.2 ± 126.6 | 73.2 ± 58.1 | 28.0 ± 23.8 | 7.2 ± 5.7 | 30.6 ± 26.0 | 25.5 ± 26.0 | 1634.4 ± 1566.0 | 2284.1 ± 1914.6 | 12450.9 ± 27341.7 | 1626.8 ± 3654.7 | 0.3 ± 0.7 | 19.4 ± 32.4 |
| *p-value*^†^ | 0.6996 | 0.0589 | 0.1855 | 0.1881 | 0.7009 | 0.0537 | 0.0621 | 0.0579 | **<0.001** | 0.4037 | 0.1014 | 0.0693 | 0.3162 | 0.7641 | 0.6586 | 0.8555 |
| Abbreviations: CHO: carbohydrates; Sat: saturated; PUFA: poly-unsaturated fat; MUFA: mono-unsaturated fat; Ca: calcium; Na: sodium; K: potassium; Vit. A: vitamin A; Vit E.: vitamin E; ^*^Differences between groups were tested using t-test; ^†^Differences between groups were tested using ANOVA test; Data are presented as mean ± standard deviation; Bold values indicate statistically significant associations (p<0.05). | | | | | | | | | | | | | | | | |

**Supplementary Table 4.** Dietary habits among the participants. Eleven food groups which follow: non-refined cereals, fruits, vegetables, legumes, potatoes, fish, meat and meat products, poultry, full-fat dairy products, olive oil and alcohol intake.

| **Food groups** | **Overall** (N=1137) (%) |
| --- | --- |
| **Non-refined cereals** | |
| Never | 17 (1.5) |
| 1-6 portions/week | 211 (18.6) |
| 7-12 portions/week | 449 (39.5) |
| 13-18 portions/week | 389 (34.2) |
| 19-31 portions/week | 63 (5.5) |
| >32 portions/week | 8 (0.7) |
| **Fruits** | |
| Never | 14 (1.2) |
| 1-4 portions/week | 27 (2.4) |
| 5-8 portions/week | 260 (22.9) |
| 9-15 portions/week | 468 (41.2) |
| 16-21 portions/week | 303 (26.6) |
| >22 portions/week | 65 (5.7) |
| **Vegetables** | |
| Never | 23 (2.0) |
| 1-6 portions/week | 44 (3.9) |
| 7-12 portions/week | 164 (14.4) |
| 13-20 portions/week | 446 (39.2) |
| 21-32 portions/week | 341 (30.0) |
| >33 portions/week | 119 (10.5) |
| **Legumes** | |
| Never | 28 (2.5) |
| Less than 1 portion/week | 69 (6.1) |
| 1-2 portions/week | 209 (18.4) |
| 3-4 portions/week | 593 (52.1) |
| 5-6 portions/week | 215 (18.9) |
| >6 portions/week | 23 (2.0) |
| **Potatoes** | |
| Never | 5 (0.4) |
| 1-4 portions/week | 80 (7.1) |
| 5-8 portions/week | 464 (40.8) |
| 9-12 portions/week | 471 (41.4) |
| 13-18 portions/week | 95 (8.4) |
| >18 portions/week | 22 (1.9) |
| **Fish** | |
| Never | 12 (1.0) |
| Less than 1 portion/week | 132 (11.6) |
| 1-2 portions/week | 725 (63.8) |
| 3-4 portions/week | 251 (22.1) |
| 5-6 portions/week | 17 (1.5) |
| >6 portions/week | 0 (0.0) |
| **Meat and meat products** | |
| 1 or less than 1 portions/week | 6 (0.5) |
| 2-3 portions/week | 64 (5.6) |
| 4-5 portions/week | 581 (51.1) |
| 6-7 portions/week | 428 (37.6) |
| 8-10 portions/week | 54 (4.8) |
| >10 portions/week | 4 (0.4) |
| **Poultry** | |
| 3 or less than 3 portions/week | 29 (2.6) |
| 4-5 portions/week | 71 (6.2) |
| 5-6 portions/week | 211 (18.6) |
| 7-8 portions/week | 519 (45.6) |
| 9-10 portions/week | 261 (23.0) |
| >10 portions/week | 46 (4.0) |
| **Full-fat dairy products** | |
| 10 or less than 10 portions/week | 8 (0.7) |
| 11-15 portions/week | 97 (8.5) |
| 16-20 portions/week | 361 (31.8) |
| 21-28 portions/week | 395 (34.7) |
| 29-30 portions/week | 217 (19.1) |
| >30 portions/week | 59 (5.2) |
| **Olive oil** | |
| Never | 166 (14.6) |
| Rarely | 43 (3.8) |
| Less than 1 portion/week | 77 (6.8) |
| 1-3 portions/week | 205 (18.0) |
| 3-5 portions/week | 293 (26.0) |
| Daily | 350 (30.8) |
| **Alcohol intake** | |
| <300 ml | 12 (1.1) |
| 300 ml | 314 (27.6) |
| 400 ml | 508 (44.7) |
| 500 ml | 251 (22.1) |
| 600 ml | 47 (4.1) |
| >700 ml or 0 ml | 5 (0.4) |
| Abbreviations: N (%): Frequency (Percentage). | |
